# A Review of Pediatric Distal Radius Buckle Fractures and the Current Understanding of Angled Buckle Fractures

**DOI:** 10.7759/cureus.24943

**Published:** 2022-05-12

**Authors:** Noah Gonzalez, Jean-Marc P Lucas, Austin Winegar, Jason Den Haese, Paul Danahy

**Affiliations:** 1 College of Osteopathic Medicine, Lake Erie College of Osteopathic Medicine, Erie, USA; 2 College of Osteopathic Medicine, Lake Erie College of Osteopathic Medicine, Bradenton, USA; 3 Department of Orthopaedic Surgery, Lake Erie College of Osteopathic Medicine, Erie, USA; 4 Department of Orthopaedic Surgery, Lake Erie College of Osteopathic Medicine, Bradenton, USA

**Keywords:** literature review, pediatric orthopedics, torus fracture, angled buckle fracture, buckle fracture

## Abstract

Distal radius buckle fractures (DRBFs) are the most common pediatric fractures and resemble the rounded portion of a Greek pillar or torus. They result from compressive forces applied to a child’s highly plastic radius. DRBFs lack cortical and physeal disruption, which makes them relatively stable. In this review, we discuss angled DRBFs, a hypothesized subset of buckle fractures that results from an off-center compressive force. Some authors refute the existence of angled DRBFs, instead proposing new criteria for DRBF classification: measuring more than 1 cm away from the physis with two to three inflection points. Without universal diagnostic criteria, misdiagnosis is common, and the utilization of flexible treatment modalities is infrequent. Rigid immobilization with short-arm casting continues to be the mainstay of treatment in clinical practice. Yet, new protocols implementing removable elastic bandages have had comparable results to casting, including reduced healthcare expenditure, less stiffness, and improved convenience and patient tolerability. Despite the discrepancies in categorizing DRBFs, complication rates remain low, and diagnostic confusion insignificantly affects clinical outcomes. Angled DRBFs have been theorized to have intraphyseal extension, making them unstable Salter-Harris fractures. Radiographic evidence supporting or denying this claim is limited. Further research is essential to determine the stability of the angled DRBF subtype and whether they should continue to be defined and managed as buckle fractures.

## Introduction and background

**Figure 1:**
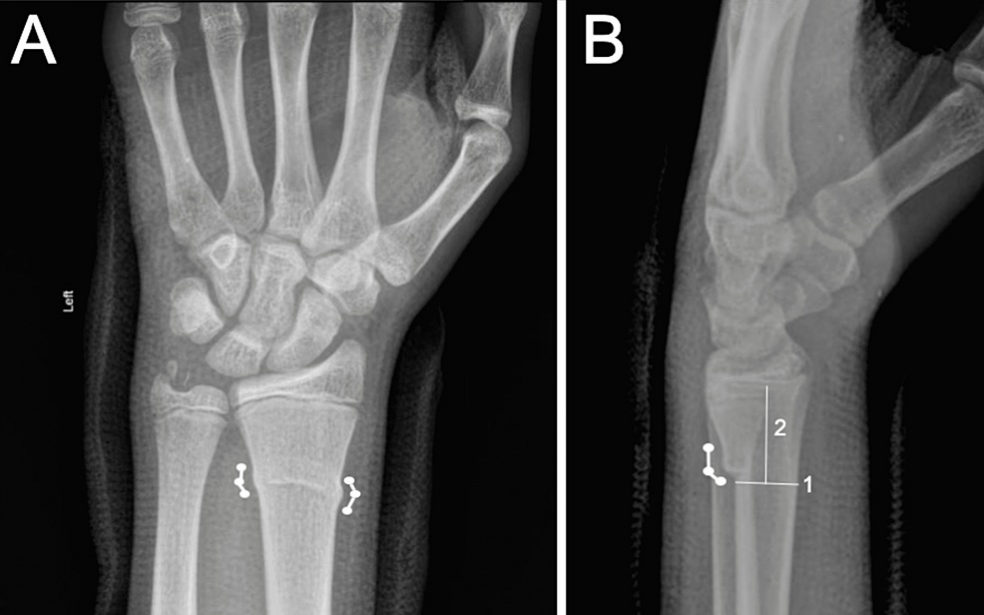
Traditional buckle fracture. Posterior-to-anterior (A) and lateral (B) X-rays of the left wrist in a 13-year-old male demonstrating a DRBF with associated ulnar styloid fracture. (A) Changes in cortical slope are noted as circles along with connecting lines (thick lines) demonstrating the path of the deformed cortical slopes. Note there are two different slopes along the path of the fracture, indicating a traditional DRBF. (B) Thin lines numbered 1 and 2 indicate how the 1-cm rule is determined. Line 1 is drawn at the proximal aspect of the fracture parallel with the distal radial physis (yellow highlight). Line 2 is drawn perpendicular to line 1 and extends to the distal radial physis. According to the rule, if the length of line 2 is <1 cm, the fracture is likely not a buckle fracture and involves the physis. Images courtesy of Dr. MT Niknejad, Radiopaedia.org, rID: 93125 [1].

Distal radius buckle (torus) fractures (DRBFs) (Figure 1) are the most frequent type of pediatric fracture and account for the highest number of fracture visits to emergency departments in the United States [1,2]. DRBFs are most commonly caused by low-energy falls on an outstretched hand, resulting in axial loading of the meta-diaphyseal junction of skeletally immature long bones [3,4]. This transition point is susceptible to failure due to the different biomechanical characteristics of the two types of bone: developing woven bone of the metaphysis and tough lamellar bone of the diaphysis [4]. When axial loads surpass the plastic deformation threshold, trabeculae fail and cause the cortex to bulge outwards at the apex of the compressive forces [5]. Buckle fractures are usually specific to children because their bone has a lower ash content (less hydroxyapatite) and is more likely to absorb force and experience plastic deformation [6]. Additionally, children have a thick periosteal sleeve above the cortex that typically stays intact and prevents unrestrained fracture extension and complete bone failure [7].

## Review

Buckle fractures and their angled counterparts

There is an active debate on what constitutes a true buckle fracture. Some authors believe the injury must convey inherent stability to be classified as a buckle fracture. Others believe that the load pattern and subsequent bone deformation delineate a buckle fracture. This ambiguity further complicates the identification of buckle fractures when there is extension into the metaphysis or when the fracture is purely unicortical [8,9]. Due to these discrepancies, the literature diagnoses DRBFs with varying degrees of accuracy. As concluded by Terreblanche et al., there is a reasonable amount of misdiagnosis: up to 46% of diagnosed DRBFs were later classified as non-buckle distal radius fractures with intraphyseal or cortical disruption [10]. This high rate of misdiagnosis implies an increasing need for a universal set of diagnostic criteria.

**Figure 2:**
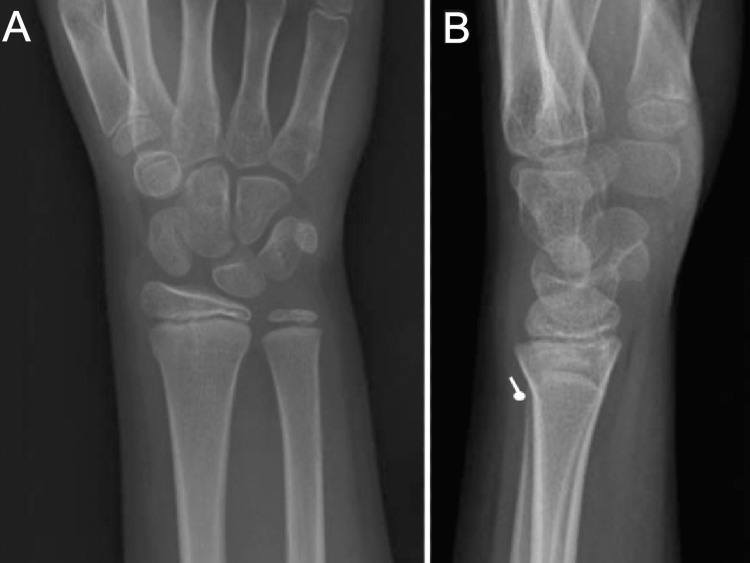
Angled buckle fracture. Posterior-to-anterior (A) and lateral (B) X-rays of a right wrist in a skeletally immature individual demonstrating an angulated DRBF. (B) Note the existence of a single cortical deflection, as is typical of an angled DRBF. Images courtesy of The Radswiki, Radiopaedia.org, rID: 12025 [11]. Note: this image was digitally modified to appear more like an angled DRBF because the original could be mistaken for a Salter-Harris. DRBF: distal radius buckle fracture

The angled DRBF (Figure 2) was first depicted by Hernandez et al. as a commonly undiagnosed derivative of pediatric DRBFs. The authors argue that a buckle fracture can exist in a secondary and more subtle form due to an unusual off-centered compression mechanism [5,11]. The phenomenon was first defined by Rogers et al. when the authors found that plastic deformation of the distal humeral metaphysis led to significant angulation accompanied by a posterior fat pad sign and without cortical disruption [12]. Angled DRBFs have also been observed in other long bones such as the distal femur and distal tibia [13]. The authors of Hernandez et al. describe this derivative as angulation of the cortex in response to the combination of the traditional axial loading with additional transverse force from either hyperflexion, hyperextension, valgus, or varus directions. The location of an angled DRBF depends on which force is being applied and can appear as an inflection point with possible extension into the physis on the dorsal, ventral, medial, or lateral aspect of the involved long bone [5]. The most common angled DRBF pattern occurs along the dorsal aspect of the distal radius and is most visible on lateral films [14]. There are numerous challenges in diagnosing an angled buckle fracture. The subtype is typically not apparent in the acute phase of injury and is usually appreciated once sclerosis saturates the fracture zone 7-10 days after injury [7]. Additionally, this type of buckle fracture typically occurs in isolation and can be easily overlooked without contralateral radiographic films or persistent soft-tissue swelling [6]. The authors believe recognition of this fracture can help differentiate it from other potentially unstable fractures and help determine fracture management [5,15].

In 2018, Iles et. al. developed the 1-cm rule to delineate DRBFs from other types of distal radius fractures, which are likely more unstable. These authors concluded that because angled DRBFs involve the distal metaphysis, they violate the 1-cm rule and thus should not be classified as buckle fractures [8]. Their logic is based on the assumption that these fracture patterns have intraphyseal extension, making them unstable Salter-Harris fractures [10]. Salter-Harris distal radius fractures are known to be more unstable than DRBFs and may necessitate operative fixation to prevent limb length discrepancies [8]. Proponents of the 1-cm rule believe a true DRBF embraces two or three demarcated inflection points that are well proximal to the physis [8,10]. Specifically, only fractures ≥1 cm proximal to the distal radial physis (Figure 1B) with less than 10 degrees of angulation and with two to three inflection points should be classified as buckle fractures [1,3,16].

Assessment and diagnosis

Initial presentations for DRBFs are no different from angled buckle fractures and include nonspecific features such as tenderness and swelling, with or without a mild deformity [8]. Physical examination may reveal decreased wrist range of motion and decreased wrist strength [17]. The standard imaging for buckle fractures is X-rays of the forearm in at least two orthogonal planes, typically with posterior-to-anterior and lateral views [18]. Some authors believe an oblique film can help accurately diagnose DRBFs because this view can help demonstrate fracture extension and cortical disruption [8]. However, other physicians advocate for fewer radiographs to reduce childhood ionizing radiation exposure [17]. The British Society for Children’s Orthopedic Surgery orders post-treatment X-rays in fewer than 17% of patients [19]. Additionally, Ling et al. found that follow-up X-rays did not change the management or outcome of buckle fractures [20]. Ultrasound (US) has been shown to be a promising alternative diagnostic tool and found in one study to be more sensitive than traditional X-rays [21,22]. US has secondary advantages for patients such as lower cost and no exposure to ionizing radiation [22]. The ongoing bedside US conducted in kids with distal upper limb fractures in the emergency department (BUCKLED) study will help determine if US is an acceptable alternative to X-rays [23]. Computed tomography (CT) and magnetic resonance imaging (MRI) are likely unnecessary for DRBF recognition due to the detection of clinically irrelevant findings, cost, and unnecessary exposure to ionizing radiation [24]. If a DRBF cannot be visualized on simple radiography or US, it is likely too small to be unstable or a threat to limb deformity.

One important limitation to Iles et al.’s study is the use of plain radiography alone. Identifying minuscule fracture lines on plain radiographs may be challenging and complicated by the presence of artifacts or skin folds. To the best of our knowledge, no studies have been conducted examining angled buckle fractures with US, CT, or MRI. MRI has been shown to be the most sensitive imaging method available for detecting trabecular derangement. Given the trabecular etiology of buckle fractures, it seems natural that future studies should incorporate MRI to evaluate for intraphyseal involvement of angled DRBFs. On the other hand, previous studies evaluating acute traumatic injuries with both plain radiograph and MRI have shown fractures on MRI in 21% to 37% of radiographs that did not show signs of fracture [25]. Thus, while it is possible that intraphyseal extension is being falsely diagnosed on plain films, it is also possible that Hernandez et al. simply missed this intraphyseal disruption. Therefore, future studies evaluating angled DRBFs with alternative diagnostic tools (eg. US, CT, or MRI) are necessary prior to passing definitive judgment on the classification of these fracture patterns.

Clinical management

Conventionally, DRBFs are treated with immobilization for two to six weeks, without the need for closed reduction [3,26]. A variety of nonoperative wrist immobilization can be used, from rigid casting to soft elastic bandage wraps [3,26]. Recently, the literature has praised more lenient treatment options that minimize patient limitations while providing equivalent immobilization when compared to below-elbow casts [22,27]. Removable splints and other flexible variations (e.g., Futura-type splint, soft bandage) have been shown to provide better mobility and quicker recovery of strength and range of motion (ROM) [18,22,26,27]. These options do not cause patients additional pain and have shown no added risk of secondary angulation or refracture at six months post-injury [27-30]. However, delayed union caused 5/90 (bandage) and 3/91 (cast) children across three studies to change immobilization device or endure a longer immobilization period [27]. This finding was statistically insignificant. Overall, patients and caregivers prefer these devices because they permit handwashing and can be removed for bathing [28]. Regardless of the treatment modality selected, it is important to avoid contact sports and activities that can lead to reinjury for six to eight weeks [18].

The authors of Hernandez et al. suggest angled DRBFs receive similar treatment as non-angled DRBFs due to their inherent stability [8]. On the contrary, if Iles et al. are correct in assuming all angled DRBFs have intraphyseal extension, they may necessitate casting, closed reduction, or operative management. Further research is needed to determine if treatment of angled DRBFs should reflect that of traditional DRBFs or Salter-Harris fractures. If present, physeal extension may be negligible, permitting the use of removable splints or elastic bandages. Until future studies definitively identify the relationship between angled DRBFs and the distal physis, we suggest clinicians avoid close reducing these fractures to avoid iatrogenic fracture propagation through the physis. Such propagation may be catastrophic relative to the initial fracture, necessitating longer periods of immobilization if not surgical fixation. Even if angled DRBFs are as stable as traditional DRBFs, circumferential casting is more appropriate in non-compliant or more active children prone to reinjury [22]. Currently, there are no specific radiographic tolerances mentioned in the literature for which non-operative DRBF management modality is indicated (e.g., soft bandage, splinting alone, or casting). Accordingly, we recommend that clinicians use their best judgment when managing these fracture patterns and assess the patient’s and caretaker’s likelihood of compliance.

**Figure 3:**
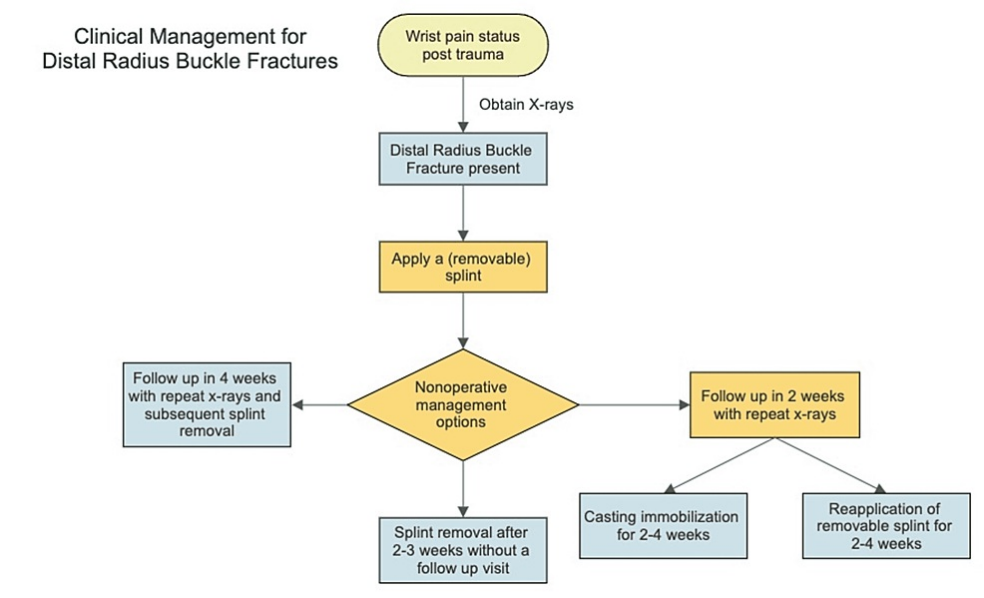
Clinical management flowchart. A descriptive summary of the current state of the literature regarding DRBF clinical management. The current gold standard of treatment follows the yellow path. DRBF: distal radius buckle fracture

The flexible treatment options such as bandages and removable splints may eliminate the need for an office visit for splint or cast removal [28]. These devices can be placed in the emergency department and removed conveniently at home two to three weeks post-injury [31]. One study found that follow-up with an orthopedic surgeon may not be necessary, so long as parents are aware of situations when medical intervention may be essential and given proper instructions on immobilization device removal at three weeks post-intervention [29]. However, because misdiagnosis is common, some authors recommend following up with an orthopedic surgeon so malunions and nonunions can be identified and treated accordingly [26,30]. West et al. suggested one follow-up visit at four weeks to be a reasonable compromise when using removable and flexible immobilization, such as soft bandages [26]. Additionally, higher-risk patient populations such as children younger than three years old, both bone fractures, pathological fractures, those with systemic disease, or those with a history of wrist surgery should have at least one follow-up appointment [8]. Figure 3 is a flowchart summarizing current treatments noted in the literature for DRBFs. In general, until diagnostic methods become more accurate, we recommend clinicians have one follow-up two weeks post-injury and another at the time of immobilization device removal.

Despite rampant misdiagnosis, long-term complication rates following DRBF treatment are paradoxically low [3]. A child that is younger than eight years old distal radius can tolerate a fracture with up to 20 degrees of dorsal angulation while still having favorable clinical results overall [7,32,33]. This implies that DRBF misdiagnosis may be clinically insignificant and even the most displaced distal radius fracture patterns may be inherently stable [2]. The authors of this review suggest that if clinicians cannot distinguish a DRBF from another type of distal radius fracture on imaging, the fracture is likely stable enough to heal with removable splinting and perhaps softer devices.

Ultimately, there is a disconnect between the studies of treatment and what occurs in practice. Despite the literary support for minimalistic treatment, only 29% of orthopedic surgeons of the Pediatric Orthopedic Society of North America treat DRBFs with removable splints. One author cites that reluctance to use more flexible management options arises from concerns about patient compliance, complications, and malpractice litigations [34]. Additionally, some parents prefer the traditional rigid form of immobilization via a short-arm cast and may feel like their child is undertreated with the flexible option or at risk for re-injury [26,30]. To the best of our knowledge, no similar studies have been conducted examining treatment preferences of angled buckle fractures. Future studies examining treatment preferences and outcomes may elicit information pertaining to the stability of these fracture patterns.

## Conclusions

Pediatric fracture experts must determine if DRBFs should be defined based on their inherent stability, the mechanism that it occurs in, or a combination of both. This will help validate future research and reduce the likelihood of misdiagnosis with an unstable distal radius counterpart that may be prone to malunion and limb deformity. There is ample literary evidence supporting the use of flexible and removable immobilization, ultrasound, and fewer follow-ups. However, barriers to traditional management reform include the risk of child or parent noncompliance with secondary malunion or nonunion and possible litigation. Ultrasound shows promise as an ionizing radiation-free imaging modality with high sensitivity for detecting DRBFs. Conservative treatment (elastic bandage or removable splints) has shown improved outcomes over traditional management, such as increased patient comfort, less short-term disability, and less healthcare expenditure. The literature has shown that treating DRBFs without follow-up assessment is possible. However, if a patient is suspected to be particularly noncompliant, we suggest at least one follow-up visit between three to four weeks post-injury to ensure proper healing, patient and parent satisfaction, and minimize litigation risk.

Little is known about angled DRBFs. Their existence was recently refuted by an article publicizing the 1-cm rule. The authors of this review believe that the 1-cm rule lacks sufficient evidence to delegitimize angled DRBFs, but acknowledge that there is insufficient evidence supporting their existence. There is currently no available literature discussing alternate diagnostic or management strategies for these fractures. More research needs to be done on angled DRBFs to determine their stability and identify optimal diagnostic workup and management.
